# What Was George Forrest’s Plant Collection Journey like in China?

**DOI:** 10.3390/plants13101367

**Published:** 2024-05-15

**Authors:** Ke Shi, Minli Jin, Renwu Wu, Yongxi Zou, Shuai Liao, Zhoubing Xiang, Jifan Luo, Xiaoxue Zhong, Zhiyi Bao

**Affiliations:** 1 College of Landscape Architecture, Zhejiang Agriculture and Forestry University, Hangzhou 311300, China; shike916@stu.zafu.edu.cn (K.S.); zouyongxi@stu.zafu.edu.cn (Y.Z.); 2022105041013@stu.zafu.edu.cn (Z.X.); luojifan553@stu.zafu.edu.cn (J.L.); zhongxx@stu.zafu.edu.cn (X.Z.); 20070007@zafu.edu.cn (Z.B.); 2 South China Botanical Garden, Chinese Academy of Sciences, Guangzhou 510650, China; shuai.liao.cn@gmail.com

**Keywords:** specimen collection, *Rhododendron*, plant collection history, ethnobotany

## Abstract

Since the 16th century, Western countries have conducted extensive plant collections in Asia, particularly in China, driven by the need to collect botanical resources and foster academic development. These activities have not only significantly enriched the Western botanical specimen collections but have also had a profound impact on the development of related disciplines such as botany, ecology, and horticulture. During this process, a large number of renowned plant hunters emerged, whose discoveries and contributions are still remembered today. George Forrest (1873–1932) was one of these distinguished plant hunters. From 1904 to 1932, he visited China seven times to collect plants and became famous for the regional distinctiveness of the species he collected. However, due to the lack of systematic collection, organization, and analysis of specimens collected by Forrest, only a few species, such as the species *Rhododendron*, are well-known among the many species he introduced to the West. Furthermore, the personal collecting characteristics and the characteristic species collected by Forrest are also not clear. This limits a comprehensive understanding of the specimen collection history and impact of Forrest in China. Therefore, systematic organization and analysis of Forrest’s plant specimens collected in China are crucial to understanding his impact on botanical classification, *Rhododendrons* introduction, global horticulture, and plant propagation. This study aims to systematically organize and analyze the plant specimens collected by George Forrest in China to investigate the family, genus, and species composition of the collected specimens and the seven collection expeditions of Forrest in China, as well as the time and altitude of these collections. Furthermore, it seeks to discuss Forrest’s scientific contributions to the global spread of plants, the widespread application of the *Rhododendron*, and his impact on the development of modern gardens, providing a theoretical basis and data reference for related research and professional development. To this end, we extensively consulted important historical literature related to Forrest and systematically collected data from online specimen databases. The conclusions drawn from the available data include 38,603 specimens, with 26,079 collection numbers, belonging to 233 families, 1395 genera, and 5426 species, which account for 48.24%, 32.63%, and 14.17% of the plant families, genera, and species in China, respectively. *Rhododendron* specimens made up 17.20% of the specimens collected in this study. The collection locations cover three provinces or autonomous regions, 11 prefecture-level cities, and 25 counties. Furthermore, we found that Forrest’s collections were concentrated in spring and summer, mainly in high-altitude areas, with 135 species found below 1500 m and 3754 species at 1500 m and above. *Rhododendron* specimens were mostly found above 3000 m.

## 1. Introduction

Western developed countries have extended their research on plant resources far beyond their own borders [1,2], making plant specimens from various national herbaria important reference materials for botanical research. Plant specimen collection is closely related to ethnobotany, which is concerned not only with the classification and usage of plants but also with the interactions between plants and local cultures, traditional knowledge, and social practices. The collection and study of plant specimens provide an empirical basis for ethnobotany, enabling researchers to gain a deeper understanding of the history of plant usage and cultural significance in specific ethnic cultures and providing important research materials for understanding the corresponding local ethnic cultures. Airy Grasa et al. demonstrated the robustness of pharmaceutical ethnobotanical knowledge in the Catalan region by studying about 6000 plant specimens from the herbarium of Catalan pharmacist and naturalist Francesc Bolòs (1773–1844) and concluded that the historical herbarium is a relevant source of ethnopharmacological information [3]. Ingvar Svanberg et al. explored the folkloric botanical knowledge of Loptuq by studying the plant specimens collected by the Swedish explorer Sven Hedin (1865–1952) in the early 20th century, as well as demonstrating that historical herbarium is of great value in understanding the culture and history of Loptuq [4].

China is recognized as one of the countries with the richest biodiversity [5], and it has seen its plant resources highly valued and widely utilized in Western countries [6]. This phenomenon dates back to the 16th century when Western botanists and collectors began to explore China’s abundant plant resources [7]. Over time, this botanical collection craze attracted many Western botanists and collectors to China, where they conducted extensive collection activities, significantly enriching Western botanical studies and making notable contributions to global garden landscapes and biodiversity [8,9,10]. This process has given rise to a number of renowned plant hunters whose discoveries and contributions are still remembered today [11,12,13].

George Forrest (1873–1932) was an outstanding botanist and plant hunter born in Falkirk, Scotland. He studied at Kilmarnock Academy and learned some medicinal knowledge of plants and the art of making herbarium specimens from a local pharmacist, which laid a solid foundation for his later work at the herbarium of the Royal Botanic Garden Edinburgh. Forrest played a significant role in the early 20th-century plant collection boom [14]. From 1904 to 1932, he made seven collecting expeditions in China, visiting areas in Yunnan Province, Sichuan Province, and the Tibet Autonomous Region, and over 100 species have been named after him [15], making him one of the most famous plant hunters in the 20th century. Forrest was renowned for collecting species with distinctive regional characteristics, such as those from *Rhododendron*, *Primula*, and *Gentiana*. These species, which have been continuously propagated and bred into new varieties in Western gardens, owe much to his extensive collections in China [16,17,18].

Forrest was not only a plant collector but also a practitioner of ethnobotany. During Forrest’s plant collecting expeditions, he not only traveled deep into the mountains and rivers of Southwest China but also established good relationships with local residents. Through his interactions with them, Forrest not only learned about the local language and customs but also gained first-hand knowledge of the unique plant culture of Southwest China. He provided his local assistants with in-depth training in plant collection and classification, imparting botanical knowledge while assimilating local understanding and use of plants. Years of exploring China gave Forrest a deep insight into the natural geography, biodiversity, and ethnic cultures intertwined with them in Southwest China. The plant specimens he collected not only recorded the botanical diversity of Yunnan and its surrounding regions but also served as a valuable source of ethnobotanical research. These specimens not only reveal the biological characteristics of the plants but also contain a wealth of knowledge about the local people’s perception, utilization, and conservation of these plants. Therefore, the study of Forrest’s botanical specimens is of inestimable value to the in-depth understanding of botanical culture, history, and the role of plants in the local society in Southwest China.

Although Forrest’s collecting activities hold a very important position in the history of botany, existing research on his plant specimen collections in China is scarce and mainly based on historical literature, lacking substantial data sources for support. There is also a deficiency in research and analysis on factors such as the collection time and altitudes of the plant specimens, which affects the accuracy and comprehensiveness of the studies, limiting our understanding of Forrest’s history and impact of plant specimen collection in China [19]. Therefore, there is a need to systematically collect, organize, and study the plant specimens of Forrest in China to fill the gaps in current research. We extensively reviewed important historical literature related to Forrest and utilized online databases of herbarium collections. We employed Python to process data information, visualized the results of his collections, and analyzed his seven expeditions in China, including the species collected, date of collection, regional distribution of the collected plants, and factors related to the habitats, seasons, as well as preferences of the collected species. The study aimed to thoroughly review Forrest’s botanical collections in China, explore his contributions to global plant dissemination, assess his impact on modern garden development, as well as provide a theoretical basis and data reference for related research.

## 2. Data and Methods

### 2.1. Data Sources

The sources of specimen data for this study are primarily divided into two parts: (1) online databases of collections held by various countries, as detailed in Table 1; (2) literature sources, as detailed in Table 2. The data collection cut-off date was 30 August 2022. A total of 38,603 plant specimen records were collected in this study, with each specimen record containing the collection number, species name, date of collection, place of collection, and some other information from the original collection record.

### 2.2. Data Processing

#### 2.2.1. Preprocessing

To address inconsistencies in data sources, formats, and completeness, we used a combination of Python programming and manual processing for data preprocessing. The original specimen collection records contained non-standardized date entries such as “19050800”, “15-Nov-05”, “1906/2/9”, and so on. To standardize date formats, we first reviewed the collection dates in the specimen data, converting various record formats into corresponding regular expressions for extracting the year, month, and day, and standardized the dates to the ‘year/month/day’ format. For date formats that the program could not automatically process, we manually standardized these dates. Similarly, the original altitude records were not standardized, with entries like “6000 to 7000 ft”, “6000–7000 ft”, “9–10,500 ft”, and so on. For the automated processing of altitude data, we adopted a similar approach: first reviewing the specimen data for collection altitudes, converting various record formats into corresponding regular expressions for extracting altitude data, and converting the altitudes to meters in a standardized format. For altitude data that the program could not automatically extract and process, we manually handled the extraction and processing of these altitudes. These unextractable altitude data often stemmed from spelling errors in the original records, such as ‘0’ being written as the letter ‘o’, which significantly increased the difficulty of data preprocessing.

#### 2.2.2. Scientific Name Verification

In order to verify the scientific names of the specimen data, we used the plant name verification function provided by iPlant (https://www.iplant.cn/pnc, accessed on 3 December 2023) along with the World Checklist of Selected Plant Families (https://wcsp.science.kew.org/, accessed on 3 December 2023) to determine the most up-to-date taxonomic scientific names of the specimens. The plant name verification function provided by iPlant includes three modes: iPlant Name Standardization, Catalogue of Life China (CoL-China) 2022 Annual Checklist, and Global Botanical Nomenclature Verification. To ensure the accuracy and completeness of the scientific name verification as much as possible, we used these three modes in order during the verification process and supplemented them with the World Checklist of Selected Plant Families.

#### 2.2.3. Recoding Collection Numbers

Since different collections might have dissimilar identification results for the specimens with the same collection number, this study adhered to the following principles to finalize the identification results of specimens of the collection number:

- If the collection number existed in both the online data and the original literature, the latest identification results (family, genus, species) of the online herbarium and other specimen information (time and location) in the original literature were retained;
- If there were multiple specimens with the same collection number but different identification results, we followed the principle of majority rule, combining with the specimen information and expert judgment to prioritize the identification result that had the highest number of consistent identifications across different specimens, and assigned the same new collection number to these specimens. If it was not possible to determine the final result based on the majority rule, then we assigned different new collection numbers to these specimens in sequence.

#### 2.2.4. Collection Date

Since the specimen data from herbaria was obtained through the recognition of Forrest’s original handwritten information, errors inevitably occurred in this process. For some clearly erroneous or unrecognizable collection dates, we attempted to correct or complete them based on some reasonable rules. Specifically, if the collection date of a specimen was obviously outside the range of Forrest’s collecting years, we identified it as an incorrect collection date. We then corrected it based on the collection dates of other specimens with the same collection number, following the principle of majority rule. If the majority of collection dates for specimens with the same collection number were the same, we used this collection date to correct the erroneous collection date, thus ensuring the accuracy of the data. For specimens whose collection dates could not be effectively recognized, we completed them following the same principles, thereby further enhancing the completeness of the data.

#### 2.2.5. Collection Location

**Figure 1:**
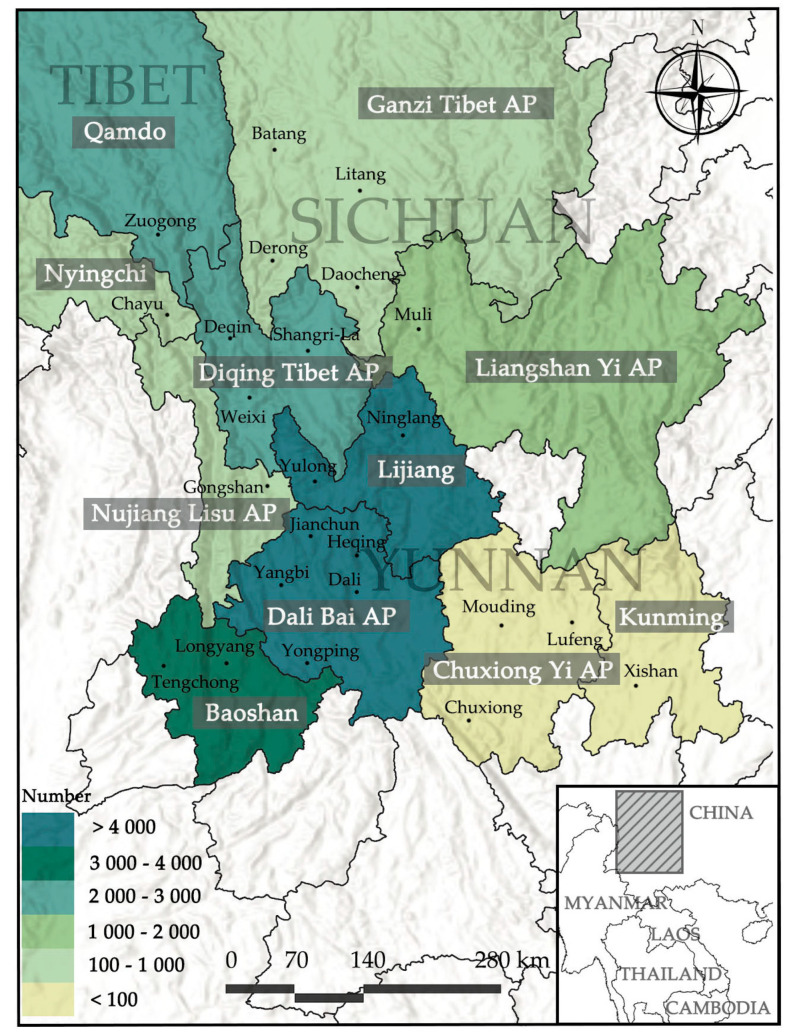
Distribution map of the collected specimen number. (AP: Autonomous Prefecture, the color on the distribution map represents the number of specimens collected by Forrest and his team, with darker color indicating a higher number of collected specimens.).

To analyze the routes and areas covered by the specimen collected by Forrest, we needed to extract the location descriptions from the original records of the specimens, which were written in the Wade–Giles system. Since the spelling of location names often incorporated dialects and were non-standard, this presents considerable challenges in verifying location names, necessitating the use of information from multiple sources. The primary sources for this study include *The Place Name of Chinese Plant Collection* [20] and internet searches. For specimens whose location names could not be extracted, we determined the specific locations of the specimens based on the latitude and longitude information from the original records. Subsequently, we further investigated changes in the administrative divisions of China, comparing ancient maps from the literature [21] with current administrative maps to definitively determine the municipal affiliation of the collection locations. Once the location names were established, they were combined with the number of specimens collected from each location and a region heatmap of the collected specimen number (Figure 1) was created.

## 3. Results of Analysis of Forrest’s Specimen Collection Activities

### 3.1. Taxonomic Composition

#### 3.1.1. Family, Genus, and Species

According to the collected data, there are 38,492 identified plant specimens belonging to 233 families. In terms of the number of collected specimens (Table 3), the top 10 families are Ericaceae (7426 specimens), Rosaceae (2672 specimens), Asteraceae (2294 specimens), Primulaceae (2031 specimens), Orchidaceae (1640 specimens), Fabaceae (1288 specimens), Ranunculaceae (1200 specimens), Lamiaceae (799 specimens), Gentianaceae (682 specimens), and Papaveraceae (595 specimens). Looking at the number of species collected (Table 4), the top 10 families are Asteraceae (478 species), Rosaceae (335 species), Ericaceae (323 species), Fabaceae (230 species), Orchidaceae (229 species), Lamiaceae (205 species), Primulaceae (178 species), Ranunculaceae (173 species), Gentianaceae (116 species), and Orobanchaceae (108 species). It is evident that, whether from the perspective of the number of specimens or the number of species collected, Forrest’s collection obviously favored species with ornamental value, such as those from Ericaceae, Rosaceae, and Asteraceae.

According to the collected data, there are 38,440 identified plant specimens belonging to 1395 genera. In terms of the number of collected specimens (Table 5), the top 10 genera by collection number are *Rhododendron* (6640 specimens), *Primula* (1488 specimens), *Cotoneaster* (425 specimens), *Gentiana* (419 specimens), *Saxifraga* (373 specimens), *Euonymus* (363 specimens), *Meconopsis* (346 specimens), *Pedicularis* (345 specimens), Sorbus (339 specimens), and *Rubus* (338 specimens). In terms of the number of collected species (Table 6), the top 10 genera are *Rhododendron* (242 species), *Primula* (103 species), *Pedicularis* (85 species), *Gentiana* (70 species), *Saussurea* (67 species), *Saxifraga* (63 species), Rubus (51 species), *Cotoneaster* (51 species), *Corydalis* (41 species), and *Ligularia* (39 species). It is evident that Forrest had a particular interest in flowering plants such as those from the *Rhododendron*, *Primula*, and *Gentiana*, which can be extensively used in horticulture.

According to the collected data, there are 36,336 identified plant specimens belonging to 5426 species. In terms of the number of collected specimens (Table 7), the top 10 species by collection number are *Rhododendron rupicola* (204 specimens), *Rhododendron beesianum* (145 specimens), *Rhododendron fulvum* (135 specimens), *Rhododendron primuliflorum* (128 specimens), *Rhododendron roxieanum* (121 specimens), *Rhododendron anthosphaerum* (119 specimens), *Rhododendron wardii* (113 specimens), *Rhododendron haematodes subsp. chaetomallum* (99 specimens), *Rhododendron adenogynum* (98 specimens), and *Rhododendron racemosum* (97 specimens). It is evident that Forrest frequently collected the species he favored, particularly various species of *Rhododendron*.

#### 3.1.2. Species Composition of Main Genera

Among the specimens collected by Forrest, the main genera with higher numbers and greater influence on later generations are *Rhododendron*, *Primula*, *Gentiana*, and *Cotoneaster*. Forrest collected 242 species of *Rhododendron*, among which there is only 1 species with over 200 specimens, accounting for 0.42% of the *Rhododendron* species, and 6 species with 100–199 specimens, making up 2.47% (Table 8). He collected 103 species of *Primula*, with 4 species having 40 or more specimens, representing 3.88% of the *Primula* species, and 9 species with 30–39 specimens, accounting for 8.74% (Table 9). Seventy species of *Gentiana* were collected by him, with two species having over 20 specimens, comprising 2.85% of the *Gentiana* species, and nine species with 10–19 specimens, representing 12.86% (Table 10). In addition, Forrest collected 45 species of *Cotoneaster*, with 8 species having over 20 specimens, making up 17.78% of the *Cotoneaster* species, 6 species with 10–19 specimens, representing 13.33%, and 31 species with 1–9 specimens, accounting for 68.89% (Table 11).

### 3.2. Collection Expeditions

In this section, we will analyze the regions, time, and altitudes involved in the seven collecting expeditions of Forrest and his team by combining previous literature studies and corroborating with specimen information. It is important to note that Forrest rarely distinguished between plants he collected himself and those collected by his team. For example, the same plant species collected on the same day might be recorded as being collected in Tengchong (Teng Yueh) and another in Lijiang (Lichiang), likely because these specimens were collected by different people. Therefore, it is almost impossible to determine Forrest’s exact collection routes; we can only attempt to reconstruct the areas he visited as accurately as possible. Dr. J. Macqueen Cowan of the Royal Botanic Garden Edinburgh reviewed nearly all specimens and marked on a map the areas Forrest collected from during each expedition. The results indicate that Forrest preferred to repeatedly collect in important areas to avoid missing crucial information. Our data collection shows the same conclusions. Furthermore, a significant portion of Forrest’s sponsors had an interest in or requested the collection of *Rhododendrons*, making the collecting of *Rhododendrons* one of Forrest’s primary tasks.

#### 3.2.1. Collection Location

As can be seen from the distribution map (Figure 1), the areas where Forrest and his team collected plant specimens were mainly concentrated in the northwestern part of Yunnan Province, the southeastern part of the Tibet Autonomous Region, and the southern part of Sichuan Province. Among the specimen data collected for this study, specimens that could be pinpointed to a specific city based on original location records totaled 18,210, with the Dali Bai Autonomous Prefecture in Yunnan having 5012 specimens, which is the highest collected specimen data and Lijiang City having 4943 specimens. Other areas included Baoshan City with 3230 specimens, Diqing Tibetan Autonomous Prefecture with 2147 specimens, Nujiang Lisu Autonomous Prefecture with 253 specimens, Kunming City with 77 specimens, and Chuxiong Yi Autonomous Prefecture with 62 specimens. In Sichuan Province, Liangshan Yi Autonomous Prefecture had 1015 specimens. In the Tibet Autonomous Region, Nyingchi City had 635 specimens, and Qamdo City had 2626 specimens. In addition, the distribution map reveals that the number of specimens collected in the northwestern region is significantly higher than in other areas. The terrain of Yunnan slopes from high in the northwest to low in the southeast, descending gradually in a stair-step fashion from north to south. In the descriptions of specimen locations, Forrest often mentioned mountains, indicating that he frequently revisited higher-altitude areas in the northern region for specimen collection. This may also be related to the natural distribution of *Rhododendrons*, as Forrest noted that the variety of *Rhododendrons* increased towards the northwest. Thus, while fulfilling his sponsors’ tasks of collecting *Rhododendron* species, this process also allowed him to gather a large number of alpine flowers, including species from the *Primula* and *Gentiana*.

**Figure 2:**
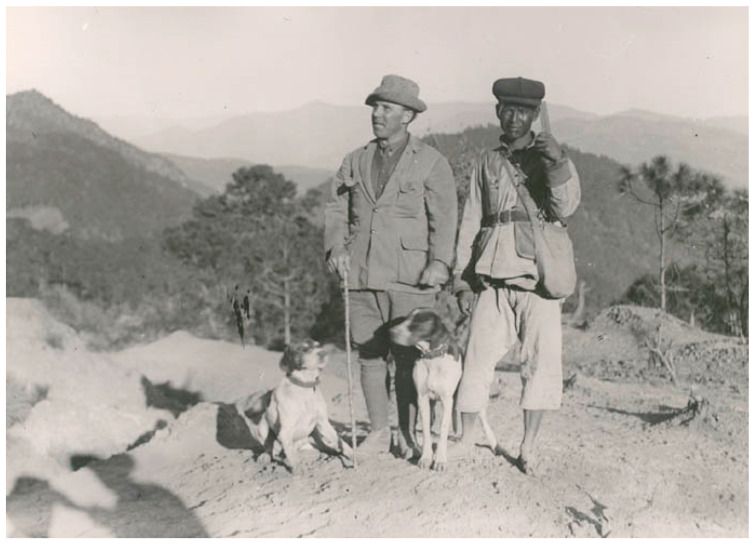
Forrest and his chief collector Zhao Chengzhang. Source: https://stories.rbge.org.uk/archives/14188 (accessed on 20 April 2024).

Forrest’s first collecting expedition spanned from May 1904 to April 1907, funded by Bees Ltd. (Cheshire, UK), the company of Arthur Kilpin Bulley. The specimens collected during this period were numbered 1–5498, and according to the data statistics, this study has collected 3957 specimen data within this timeframe. In May 1904, Forrest embarked on a cruise through the Suez Canal and Bombay, India, heading to Yangon, Myanmar. He arrived in Bhamo, Myanmar, in early July, traversed the high mountains and gorges between the Salween-N’Mai-hka (known as Nu River in China) and the Mekong River, and reached Tali-fu (including Dali, Eryuan, and Xianyun) by the end of August. In September, he traveled from Songgui (Sung Kwei) to Shangri-La (Chungtien), then crossed the Yangtze River, traversed the watershed between the Mekong and the Yangtze, and arrived at the alpine pasture of Fugong (Fu-kung). He then headed north to Cikai (Tsekou). In April 1905, he arrived in Cikai and collected for a week on the mountain 19 km northwest. In July, due to an uprising, he fled south along the west bank of the Mekong River to Weixi (Wei His), where he lost a lot of his findings along the way. In October, Forrest set out from Tengchong to collect in the upper reaches of the Nu River, crossed the watershed of the Nmai Kha-Shweli River, reached the Nujiang Lisu Autonomous Prefecture, then arrived at the watershed between the Nu River and the Mekong River, and continued westward through the Nu River valley for further collection, all the way to the source of the Longchuan River. It was difficult to find local guides to provide accurate routes and distances due to the war during this period. In March 1906, Forrest unfortunately fell ill, but his assistant continued to collect near Jade Dragon Snow Mountain, and after his recovery, he continued to collect in the Cangshan (Tsang-shan) and Jade Dragon Snow Mountain area. In 1907, Forrest returned to England, and during this journey, he met his lifelong assistant Zhao Chengzhang (Figure 2), who would help Forrest in leading the team for plant specimen collection. According to the specimen information, in addition to the locations mentioned above, Forrest or his assistants also visited Kunming City, Chuxiong Yi Autonomous Prefecture in Yunnan Province, Liangshan Yi Autonomous Prefecture, and Ganzi Tibetan Autonomous Prefecture in Sichuan Province, as well as Qamdo City in the Tibet Autonomous Region.

The second collecting expedition spanned from November 1910 to March 1911, also funded by Bees Ltd. (Cheshire, UK), the company of Arthur Kilpin Bulley. The plant specimens collected during this period were numbered from 5499 to 7401, and our study gathered data on 2677 specimens from this period. In 1910, Forrest departed from Liverpool, headed for Yangon, and reached Tengchong. He collected in the area around the watershed between the Ruili River (Shweli River) and the Nu River. In May of the same year, he encountered a memorable *Rhododendrons* bloom in Songgui and collected a large number of *Rhododendron* specimens. Additionally, the specimen information indicates that he or his assistants also reached Nyingchi City and Qamdo City in the Tibet Autonomous Region.

The third collection period was from February 1912 to March 1915. This expedition was funded by John Charles Williams, who provided Forrest with an annual salary of 500 pounds to collect plants in Yunnan for three years. The specimens collected during this period were numbered from 7402 to 13,598. Our study gathered data on 8248 specimens from this period. In February 1912, Forrest traveled from Bhamo towards Tengchong. At this time, China was undergoing a democratic revolution. In early May, Forrest and his assistants freely collected near the watershed between the Nu River and the Longchuan River, and by August 31, when an armed revolution broke out in China, they had already collected more than 1700 different plant species. In early September, he retreated to Myanmar from Tengchong to avoid the turmoil, but his assistants continued the collection. In early 1913, Forrest returned to Yunnan and conducted collections in the mountains near Dali and Lijiang. This time, he increased the number of assistants, who were dispersed throughout Yunnan to collect, significantly enhancing the efficiency of the collection. Four were at the Nu River–Longchuan River watershed, two near the Cangshan, two to four in the Zhongdian mountains 190 km northwest, and Forrest, with the remaining eight, went to the northwest of the Jade Dragon Snow Mountain. In early January 1914, Forrest returned to Tengchong to collect and sent assistants to collect upstream of the Mekong River and Baima Shan (Beima Shan), subsequently shipping back over 6000 plant specimens to England. In early January 1915, Forrest returned to England from Yangon. According to the specimen data, during this collection period, Forrest’s team also reached places including the Nujiang Lisu Autonomous Prefecture in Yunnan Province, the Liangshan Yi Autonomous Prefecture, and the Ganzi Tibetan Autonomous Prefecture in Sichuan Province, and Qamdo City and Nyingchi City in the Tibet Autonomous Region.

The fourth collection period was from January 1917 to March 1920, funded by 12 institutions or individuals. The specimens collected during this period were numbered from 13,599 to 19,333, and our study gathered data on 7569 specimens from this period. Forrest departed from Liverpool to Yunnan, establishing a collection base centered north of Cikai. He dispatched assistants to the surrounding areas, thoroughly collecting from two important regions: one was north of Chu-la at the Mekong–Salween watershed, and the other one was on the Baima Shan located at the Mekong–Yangtze watershed. This expedition collected a large number of *Rhododendrons*, and the collected materials were shipped back to England in August 1918. Unfortunately, an accident occurred during the journey, resulting in the loss of a substantial amount of materials. According to the specimen information, during this collection period, Forrest’s team also reached the Dali Bai Autonomous Prefecture, Baoshan City, and Lijiang City in Yunnan Province; Liangshan Yi Autonomous Prefecture, and Ganzi Tibetan Autonomous Prefecture in Sichuan Province; and Qamdo City and Nyingchi City in the Tibet Autonomous Region.

The fifth collection period spanned from January 1921 to March 1923, funded by Williams and Reginald Cory in England. The specimens collected during this period were numbered from 19,334 to 23,258, and our study gathered data on 4841 specimens from this period. During his collecting process heading west, Forrest crossed the watershed between the Nu River and the Dulong River. During his collecting process heading north, Forrest followed the watershed between the Mekong and Nu Rivers, passing through Eryuan, Heqing (Hoching), Weixi, Shangri-La, Muli, Lijiang, and Yongbei (Yung-Pei). Forrest returned to England in March 1923, and during his time in England, his collection team continued their work, with many specimens collected in Jianchuan (Chien Chuan). According to the specimen information, Forrest’s team during this collection period also reached Baoshan City in Yunnan Province, Ganzi Tibetan Autonomous Prefecture in Sichuan Province, and Nyingchi City and Qamdo City in the Tibet Autonomous Region.

The sixth collection period spanned from January 1924 to March 1926, with the initial funding for the expedition to China provided by Bulley and Williams. The specimens collected during this period were numbered from 24,000 to 27,833, and our study gathered data on 3780 specimens from this timeframe. Forrest traveled through the watersheds between the Nu River and the N’Mai Kha River, as well as between the Lancang River and the Nu River. In 1929, in Forrest’s absence, his team collected several hundred specimens near the Lijiang Range, with collection numbers from 27,834 to 28,361. By late September 1929, Forrest’s team had collected over 400 varieties of dried plant specimens. According to the specimen information, during this collection period, Forrest’s team also reached the Dali Bai Autonomous Prefecture, Baoshan City, Lijiang City, Diqing Tibetan Autonomous Prefecture, and Nujiang Lisu Autonomous Prefecture in Yunnan Province, as well as Nyingchi City and Qamdo City in the Tibet Autonomous Region.

**Figure 3:**
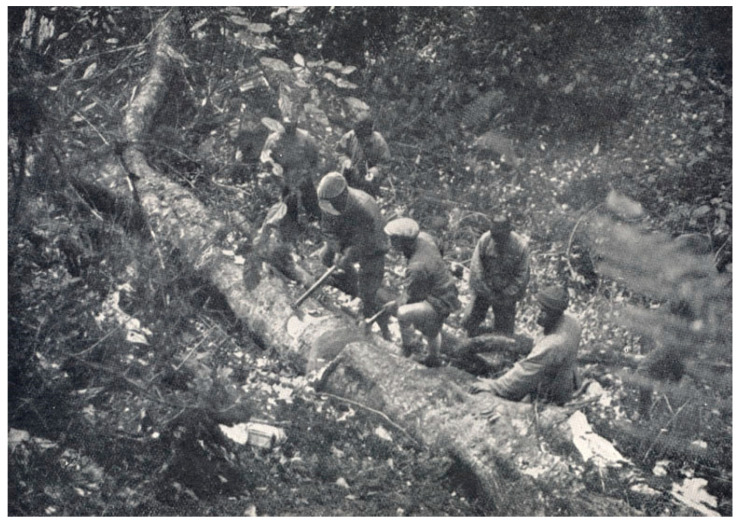
Forrest’s collectors were cutting up the massive *Rhododendron*. Source: https://stories.rbge.org.uk/archives/14186 (accessed on 20 April 2024).

The last collection period was from November 1930 to January 1932, which also marked the most heavily funded of Forrest’s seven expeditions in China, with support from 39 individuals or institutions. The specimens collected during this period were numbered from 28,362 to 31,015, and our study gathered data on 1634 specimens from this timeframe. The main objective of this collection was to find plants that had been missed in previous expeditions; thus, Forrest revisited the aforementioned areas. According to the specimen information, during this collection period, Forrest’s team reached the Dali Bai Autonomous Prefecture, Baoshan City, Lijiang City, Diqing Tibetan Autonomous Prefecture in Yunnan Province; Liangshan Yi Autonomous Prefecture, Ganzi Tibetan Autonomous Prefecture in Sichuan Province; and Changdu City in the Tibet Autonomous Region. In 1931, Forrest discovered a giant Rhododendron in the forests of the Gaoligong Mountains in Tengchong, Yunnan (Datang Village, Jietou Township, north of Tengchong), which was 25 m tall, 87 cm in diameter, and approximately 280 years old. He instructed his assistants to cut it down and secretly removed a disk from near the base as a trunk specimen (Figure 3). This specimen caused a sensation in the botanical community at the time. *Rhododendron protistum* var. *giganteum* is considered one of the species in Rhododendron with the largest tree form and flowers in the world, which also earned Forrest the title of “King of the Rhododendrons”. On 5 January 1932, he passed away unexpectedly during a plant collection trip, and his body was buried in Tengchong.

#### 3.2.2. Collection Time

**Figure 4:**
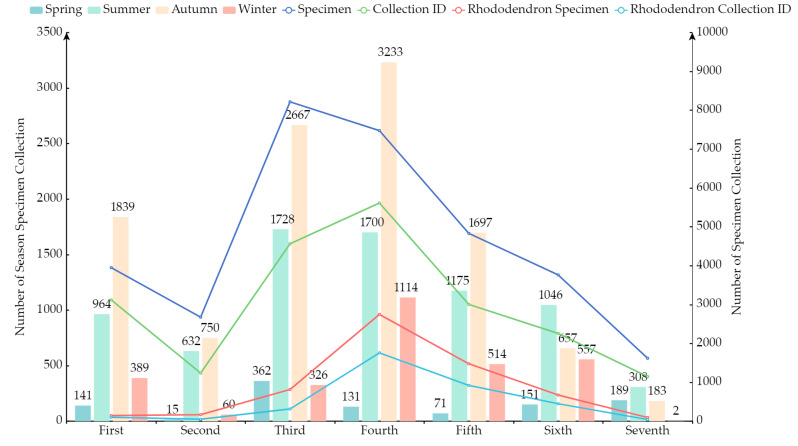
Number of specimens collected in seven collecting expeditions and their seasonal distribution. (Collection ID refers to the new collection number assigned to the plant specimens after recoding).

As can be seen from Figure 4 and Table 12, the fourth collection had the highest number of plant specimens, and the third and fourth collections had the largest number of species. The collection of *Rhododendrons* was also the most abundant in the fourth collection. In fact, it was on the basis of this collection that Professor Balfour revised the taxonomy of *Rhododendron*. Additionally, the bar chart reveals that the number of specimens collected in summer and autumn is significantly higher than in other seasons. This may be due to the climate in the Yunnan region being more suitable for plant growth during the summer and autumn seasons, facilitating the efficient collection activities of the collectors, and the natural flowering period of *Rhododendron* being from April to June. Winter saw the fewest collections due to harsh climates in high-altitude areas. The line chart indicates a significant gap between the number of specimens and the Collection ID during the third collection. This may be due to the outbreak of war in China during that time, which led to a shift in the collection method to a decentralized approach by the team, coupled with a substantial increase in the number of assistants. This resulted in the chaos of collecting multiple duplicate specimens of the same species.

#### 3.2.3. Collection Altitude

**Figure 5:**
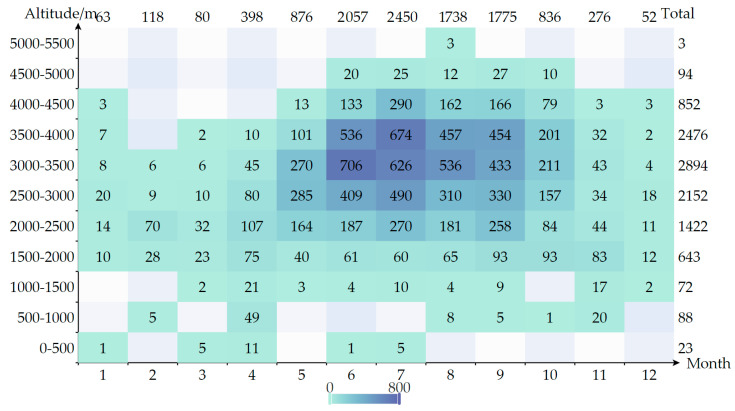
Altitude–Month Heatmap of specimen number.

**Figure 6:**
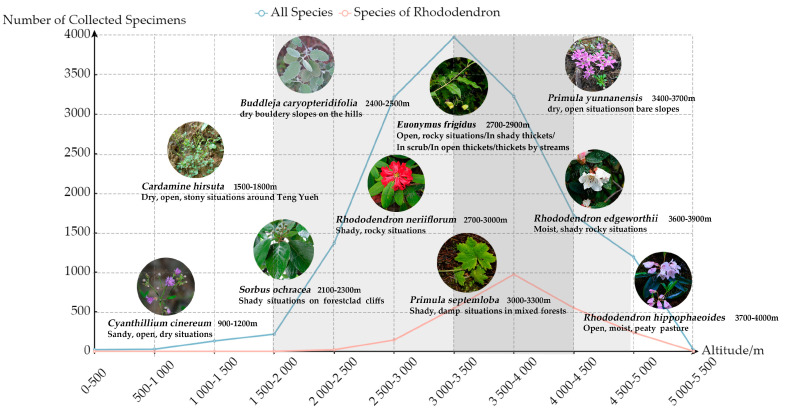
Plant altitude and habitat analysis.

As shown in Figure 5, Forrest collected the highest number of specimens in high-altitude areas in summer. The line chart in Figure 6 indicates that the number of plant specimens collected begins to significantly increase between altitudes of 2000 and 3500 m, reaching a maximum of 3975 specimens in the altitude range of 3000–3500 m. According to the specimen data collected in this study, there are 15,114 specimens with verifiable altitude data. Among these, 135 species were collected from altitudes below 1500 m, with the top 10 species by number of specimens being *Ponerorchis monantha* (10 specimens), *Bulbophyllum amplifolium* (4 specimens), *Flemingia strobilifera* (4 specimens), *Synotis saluenensis* (3 specimens), *Lycium chinense* (3 specimens), *Ainsliaea fulvipes* (3 specimens), *Chamaegastrodia inverta* (3 specimens), *Camphora tenuipilis* (3 specimens), *Hiptage acuminata* (3 specimens), and *Cleisostoma racemiferum* (3 specimens). Species collected from altitudes of 1500 m and above total 3754, with the top 10 species by number of specimens being *Rhododendron rupicola* (76 specimens), *Rhododendron beesianum* (71 specimens), *Rhododendron primuliflorum* (63 specimens), *Rhododendron fulvum* (54 specimens), *Rhododendron anthosphaerum* (53 specimens), *Rhododendron phaeochrysum* var. *levistratum* (52 specimens), *Rhododendron heliolepis* (48 specimens), *Euonymus frigidus* (48 specimens), *Rhododendron roxieanum* (42 specimens), and *Rhododendron aganniphum* (42 specimens).

The number of *Rhododendron* specimens collected from altitudes below 3000 m is 268, while those collected from altitudes of 3000 m and above total 2136. Forrest recorded that Above 1500 m, *Rhododendrons* occurred as isolated specimens or small groups growing in thickets and forests of mixed dicotyledonous shrubs and trees, gradually increasing in numbers up to 3500–4500 m, at which point they formed the dominant feature in the vegetation either as undergrowth in the forests of conifers or by themselves as dense thickets and forests. In a lecture that Forrest delivered to the members of the *Rhododendron* Society in 1920, he mentioned that except for *Rhododendron griersonianum* and *Rhododendron spinuliferum*, nearly all *Rhododendrons* in natural conditions were social plants, with isolated *Rhododendrons* being rare. In an environment where one species does not entirely dominate a situation, several or many have apparently adapted themselves to the environment for the necessary mutual protection. It was especially evident in some of the deeper side valleys of such a range as the Cangshan, where *Rhododendron brachyanthum*, *Rhododendron neriiflorum*, *Rhododendron haematodes*, and others luxuriated in the shade. Additionally, the further north or northeast, and the higher altitude, the greater the number of species. As Forrest moved northwest, he found more and increasingly magnificent *Rhododendrons*, leading him to develop a theory since 1912 that was, the real home of the *Rhododendrons* was the high alpine region on the Tibetan frontier, which formed the basins and watersheds of the Salween, Mekong, and Yangtze. There, somewhere about 98–101° E and 25–31° N, the *Rhododendrons* reached their optimum. The data in this study show that the collected *Rhododendron* specimens were also concentrated between 3000 and 5000 m, indicating that *Rhododendron* specimens collected by Forrest in the high-altitude range were particularly rich. With a few exceptions like *Rhododendron racemosum*, *Rhododendron lepidotum*, and *Rhododendron decorum*, most *Rhododendrons* exhibited distinct locality in terms of latitude, longitude, and altitude. Among the species found in the Yangtze River basin, only a few were common in the Mekong or Salween River basins, and vice versa. Additionally, in Forrest’s records, there are some descriptions of the collection environment (Figure 6) that provide valuable references for studying the living environment and habits of the species collected before.

Additionally, Forrest’s fewer collections at lower altitudes may be related to Yunnan’s unique terrain. The upstream valleys above 3500 m in Yunnan had relatively large undulations and were easily accessible once a sufficient altitude is reached, whereas the downstream valleys were rugged gorges. Forrest mentioned in his notes that collecting plants in upstream valleys might be quite straightforward, but traveling from one watershed to another could take several days.

## 4. Discussion

### 4.1. The Excellence of Forrest

As a plant collector, Forrest’s achievements stand out in several aspects, and his work not only advanced botany but also had a profound impact on the fields of horticulture, ecology, and plant conservation. Statistical analysis from the collected specimens shows that there are 115 species named after George Forrest (Supplementary Materials). Even more remarkable is the fact that species collected by Forrest received the Award of Merit (AM) from the Royal Horticultural Society (RHS) 49 times, involving 726 specimens in this study. In addition, those species collected by Forrest have received the First Class Certificate (FCC) from RHS 12 times, involving 303 specimens. These impressive numbers not only demonstrate the widespread recognition of his collecting work but also serve as strong evidence of his outstanding contributions to the field of botany.

Compared to other collectors, Forrest’s collection work was primarily concentrated in Yunnan and its surrounding areas. This geographically focused strategy reflects his in-depth study of plant diversity and efficient collection in specific regions [22,23,24]. The Yunnan region, with its unique geographical and climatic conditions, is rich in plant resources, especially species of *Rhododendron*. Forrest’s focused collection here showcases his professional skills in identifying and collecting rare plants [25,26,27]. His collection activities not only enriched the botanical knowledge base but also raised awareness and conservation efforts for endemic plants in the region, providing a crucial basis for plant conservation work. By conducting in-depth collections in specific areas, Forrest was able to frequently revisit and monitor the growth conditions of plants, obtaining more accurate data and high-quality specimens, thereby also advancing local botanical research and laying the foundation for later ecological conservation and sustainable use.

Furthermore, Forrest not only excelled in the practice of plant collecting but also showed innovation and foresight in the methods of introduction. He pioneered the integration of commercial approaches into the field of plant collecting, a transformation that not only brought new funding sources for plant research but also set a new standard for the business model of plant collecting [28]. At that time, plant collectors typically relied on sponsorship from scientific associations or government departments, but Forrest was able to attract support from individuals and private institutions, reflecting his influence in the botanical community and the appeal of his collecting work. His collecting activities were funded by more than 50 institutions or individuals, which was an unprecedented achievement at that time. It not only indicates that his collecting work was widely recognized but also suggests that his collecting strategies and outcomes held significant value for the botanical and horticultural communities [29,30]. Forrest’s innovative spirit and fundraising capabilities are an important mark of his status as an excellent plant collector and provide a new mode of collecting work and funding sources for subsequent plant collectors.

### 4.2. Collecting Preferences and the Value of Journey Recording

From the data analyzed in this study, it can be seen that Forrest preferred to collect plant specimens during the mild spring and summer seasons and primarily focused on high-altitude areas ranging from 2500 to 5000 m. In his early collections, he chose a wide variety of plants but gradually began to pay more attention to trees and shrubs, especially the species of *Rhododendron*, *Primula*, and *Gentian*. He had a particular preference for flowering species with bright colors, and he used to collect multiple specimens of the same species, which were repeatedly collected at the same locations to carefully prevent the omission of important information. In addition, the assistants he dispatched were professionally trained, which also contributed to the completeness and professionalism of his collection work. Therefore, excluding herbaceous and mossy families with inconspicuous flowers or native plants from warmer regions below 1500 m, Forrest collected flowering plants from almost all the regions he visited. In addition, we find that Forrest’s collecting seems to favor plant species with high ornamental properties. Such collecting preference was not only driven by Forrest’s personal preferences but rather based on an awareness of the potentially great economic value of the newly discovered ornamental plant species in the horticultural industry. The discovery of new ornamental plant species can stimulate widespread interest and demand in the market, thus having a profound impact on the horticultural business sector. Forrest’s collecting preference was also closely linked to his funding sources. Since his collection activities were primarily sponsored by garden owners and horticultural businesses, his efforts naturally tended to focus on ornamental plants that could bring direct economic returns to his sponsors. This strategy not only ensured ongoing support for his collecting work but also meant that his discoveries could meet the horticultural market’s constant demand for novel and attractive plants.

Although he did not publish a botanical monograph, the valuable photographs and collection notes he left behind have provided significant assistance for various studies in the southwestern region of China [31,32]. Forrest’s collecting activities not only recorded the species of plants but also their geographical distribution. These records are of great importance for understanding the distribution patterns and geographical changes of plants in the southwestern part of China. Additionally, in his plant specimen collection records, not only was there information about the altitudes where the specimens were collected, but there were also descriptions of the habitats where the plant specimens were collected. For example, in the original record of *Euonymus frigidus*, Forrest described it as “Open, rocky situations in side valleys on the eastern flank of the Lichiang Range” and “Side valleys on the mountains east of Yungning, thickets by streams”. As for *Rhododendron hippophaeoides*, Forrest’s original record states, “Open, boggy pasture on the western flank of the Lichiang Range” and “Open, moist, peaty pasture on the Haba Range”. These detailed habitat records provide important reference material for later studies on the growth habits of plants.

**Figure 7:**
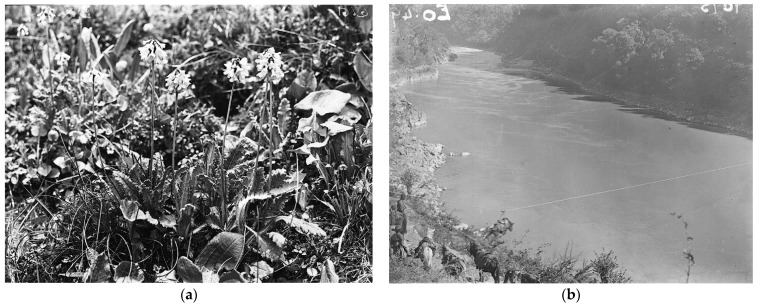
Photos taken by George Forrest: (**a**) *Primula pinnatifida* photo taken by George Forrest in 1910. Source: https://stories.rbge.org.uk/archives/14301, accessed on 24 April 2024. (**b**) A mule crossing the Cikai ropes. Source: https://stories.rbge.org.uk/archives/14301, accessed on 24 April 2024.

Furthermore, at that time in Yunnan, due to limited conditions, missionaries did not have cameras to record what they found. As a result, Forrest became one of the early collectors who used cameras to record the natural scenery and biodiversity of Yunnan. His camera work not only captured the rich and diverse plant communities of the Yunnan region but also recorded the local wildlife, magnificent natural landscapes, and the daily life of the local residents (Figure 7). Forrest’s camera work provided us with a precious historical record, allowing us a glimpse into the Yunnan of that era, with its unique culture and natural environment, and also offering valuable reference for subsequent collectors, scientists, and historians.

### 4.3. Impact on World Horticulture and Plant Propagation

Forrest’s collecting work had a significant impact on the development of world horticulture. Through his observations and studies of the natural habitats of *Rhododendrons*, Forrest proposed new insights into their growth substrates [21]. He discovered that in Yunnan, many *Rhododendron* species roots were embedded in the crevices of limestone cliffs and boulders or in the limestone gravel at the base of these formations. For the taller tree species, although humus provided support, their roots were similarly situated, or at least covered, or in contact with limestone. This discovery was groundbreaking for the prevailing horticultural practices at that time as it challenged the traditional botanical understanding of *Rhododendrons*’ growing environments. Forrest’s findings were not only significant for the theoretical development of horticulture but also had a direct impact on practical *Rhododendron* cultivation methods. By bringing limestone samples back to England, he demonstrated the natural growth substrate of *Rhododendrons* to the horticultural community, thus promoting the use of calcareous soil in *Rhododendron* cultivation practices. This change was fundamental in improving the cultivation success and healthy growth of *Rhododendrons* in England and other regions.

Forrest’s collection work not only enriched the botanical knowledge base but also fostered innovation and development in horticultural techniques. His practices and discoveries provided valuable experience and guidance for subsequent gardening enthusiasts and professionals, making the cultivation methods for *Rhododendrons* more scientific and effective. Thus, Forrest’s work left an indelible mark not only in the field of botany but also in horticultural practice. Moreover, Forrest’s collection efforts significantly influenced world horticulture and had profound impacts on plant dissemination. Western plant hunters like Forrest, who introduced Chinese plants, greatly enriched the botanical diversity of England. Previously, the total number of plant species in England was only about 1500. These hunters brought back countless rare Chinese plants, and these large-scale new discoveries influenced the direction of development for English botanical gardens and horticulture, bringing substantial economic benefits and causing a revolutionary impact on gardens in England and across the West [33]. The discovery and introduction of these rare plants changed the distribution of plants worldwide, advancing global biology and making a tremendous contribution to plant dissemination in China and globally. His efforts not only enriched the plant resources of Western countries but also advanced botanical research and horticultural practice, allowing many rare and beautiful plants to be appreciated and studied worldwide [34]. His work not only deepened Western scholars’ understanding of the plant diversity in Southwest China but also greatly enriched the botanical resources of Western countries through the numerous seeds, roots, and live plants he brought back to England [35].

### 4.4. Contribution to Rhododendron Introduction and Research

Forrest’s collection work greatly enriched the *Rhododendron* resources not only in England but also globally. For example, he discovered famous species such as *Rhododendron griersonianum*, *Rhododendron racemosum*, *Rhododendron haematodes*, *Rhododendron floccigerum*, *Rhododendron hippophaeoides*, and so on.

The discovery and introduction of these new *Rhododendron* species profoundly impacted the horticultural world in England and worldwide. The moist and mild environment of England provided the ideal conditions for the cultivation and growth of *Rhododendrons*, making them rapidly popular among gardening enthusiasts and landscape designers. For instance, the species *Rhododendron griersonianum* he introduced in 1917, as a direct parent of 159 garden hybrids, had 34 descendants that received RHS horticultural awards. Moreover, due to the large volume of seeds collected by Forrest and the numerous hybrid offspring with excellent ornamental effects, thousands of seedlings were cultivated, forcing cultivators to expand their original garden areas and even move into woodland areas, thereby influencing policies and giving rise to modern woodland gardens. Thus, it is no exaggeration to say that Forrest played at least an indirect but significant role in the evolution of modern gardens. Statistical analysis of plant specimen data reveals that Forrest collected 6640 *Rhododendron* specimens, including 242 species, not only in large numbers but also covering a comprehensive range of species. Among the *Rhododendron* specimens he collected in China, over 400 were described as new species by researchers, and more than 150 of these are still accepted by many researchers today [18]. The *Rhododendron* specimens collected by Forrest are mainly preserved in the Royal Botanic Garden Edinburgh, making it the research center with the largest collection of *Rhododendron* specimens and helping English researchers take a leading position in the taxonomic study of *Rhododendron* [36]. Many types of specimens provide important materials and evidence for research in *Rhododendron* taxonomy and phytogeography in England.

### 4.5. The Negative Impact of Forrest’s Collection Activities

Forrest’s collecting activities can be seen as the forced export of Chinese species, and the negative impacts on the development of Chinese biology and the destruction of natural resources are evident.

In the late 19th and early 20th centuries, China had not yet established a modern biodiversity conservation system. At that time, society and the scientific community lacked awareness of the importance of species protection, and there were no corresponding laws and protection measures. This led to the unrestricted collection of a large number of plant specimens, and many seeds and specimens of rare and unique species were taken overseas. A large number of type specimens and original literature were scattered in major Western research institutions, making it very difficult for Chinese researchers to study and identify species [37]. Additionally, due to Forrest’s limited knowledge of Chinese location names, lack of detailed records of plant collecting activities, and the employment of a large number of non-professional local staff, it becomes more difficult to study Forrest’s plant specimen collection in China.

During Forrest’s expeditions, sometimes, in order to reach deeper locations to collect rare or valuable plants or to hasten their expeditions, some natural resources were damaged. As Forrest and his assistants made their way through the thorn-filled valleys, they split miles of Rhododendrons because the bushes were so dense that it was difficult for them to go on the expeditions, which caused some damage to the primitive plant resources and the natural environment [21]. Another example is that after he found the world’s tallest and largest known *Rhododendron protistum* var. *giganteum* in the Gaoligong Mountains of Tengchong, Yunnan, he cut it down and brought it back to England, which was destructive and plunderous. Cutting down the giant Rhododendron without due consideration of the local community and cultural values was morally and ethically controversial. Such a behavior is disrespectful of natural resources and disregardful of the protection of ancient trees and rare plant resources.

Although Forrest’s collection activities might have contributed to science, they set a bad precedent in terms of ecological protection and sustainable development. Such actions could damage China’s image in the international community, which is seen as lacking awareness of local biodiversity protection. This also reminds us that when conducting scientific research and resource utilization, we must adopt a responsible attitude and ensure that activities conform to the principles of ecological protection and sustainable development.

### 4.6. Specimens Digitization and Information Sharing

Digitization and information sharing of plant specimens is a key direction in contemporary botanical research. It not only ensures the long-term preservation of valuable plant specimen data but also significantly enhances the usability and accessibility of the specimen data [38,39,40]. However, when processing the collected specimen data from Forrest, we faced a series of challenges, particularly in data standardization. Although original records contain information about the date, altitude, and location of collected specimens, they are not always standardized, which greatly increases the difficulty of data processing. For instance, the data format of recording specimens at different times and places may vary, and even specimens collected at the same period may differ due to the personal habits of the recorders. The altitudes may be recorded in various units, and the location information might lack precise geographic coordinates or use different place names. These issues not only pose obstacles to data integration and analysis but also affect the accuracy and reliability of the data. Therefore, the digitization process requires cleaning and standardizing these non-standard data, which could include unifying altitude units and date formats, updating place names, supplementing missing geographic coordinates, and so on. Moreover, establishing a standardized recording and data entry process is crucial for future plant specimen collecting work, which not only helps improve data quality but also provides more accurate and consistent specimen data for future researchers, offering valuable experience and lessons for future botanical research and data management.

## Figures and Tables

**Table 1:** The online sources of collected specimen records.

No.	Country	Herbarium Name	Website	Herbarium Code	Number
1	UK	Royal Botanic Garden Edinburgh	https://data.rbge.org.uk/search/herbarium/ (accessed on 30 August 2022)	E	9566
2	China	Biological Collections of the Chinese Academy of Sciences	http://nbc.ioz.ac.cn/View/Home/Specimen.aspx (accessed on 30 August 2022)	PE	7751
3	USA	Smithsonian National Museum of Natural History	https://collections.nmnh.si.edu/search/botany/ (accessed on 30 August 2022)	US	4208
4	China	Herbarium of Sun Yat-sen University	/	SYS	2240
5	China	South China Botanical Garden Herbarium	http://herbarium.scbg.cas.cn/ (accessed on 30 August 2022)	IBSC	1967
6	UK	The Natural History Museum	https://data.nhm.ac.uk/ (accessed on 30 August 2022)	BM	1857
7	UK	Royal Botanic Gardens, Kew	https://apps.kew.org/herbcat/navigator.do (accessed on 30 August 2022)	K	1564
8	China	Herbarium, Kunming Institute of Botany, Chinese Academy of Sciences	http://groups.kib.cas.cn/kun/ (accessed on 30 August 2022)	KUN	1268
9	France	Muséum National d’Histoire Naturelle	https://www.mnhn.fr/fr/collection-des-plantes-vasculaires (accessed on 30 August 2022)	P	1069
10	USA	Herbarium of the Arnold Arboretum, Harvard University Herbaria	https://kiki.huh.harvard.edu/databases/specimen_index.html (accessed on 30 August 2022)	A	1014
11	China	Herbarium, School of Life Sciences, Xiamen University	/	AU	284
12	Sweden	Swedish Museum of Natural History	https://herbarium.nrm.se/ (accessed on 30 August 2022)	S	233
13	Nederland	Naturalis Biodiversity Centre, formerly Leiden University	https://bioportal.naturalis.nl/nl/result (accessed on 30 August 2022)	L	165
14	USA	Missouri Botanical Garden Herbarium	https://tropicos.org/specimen/Search (accessed on 30 August 2022)	MO	65
15	China	The Herbarium of Nanjing University	/	N	52
16	USA	William and Lynda Steere Herbarium of the New York Botanical Garden	https://sweetgum.nybg.org/science/vh/ (accessed on 30 August 2022)	NY	45
17	Germany	Botanischer Garten und Botanisches Museum Berlin	https://www.bgbm.org/biodivinf/projects/digitalisierung/default.htm (accessed on 30 August 2022)	B	40
18	China	Herbarium of the Jiangsu Province and Chinese Academy of Sciences, Institute of Botany	/	NAS	31
19	Sweden	Museum of Evolution	https://databas.evolutionsmuseet.uu.se/botanik/home.php (accessed on 30 August 2022)	UPS	31
20	USA	The Botanical Collection at the California Academy of Sciences	https://www.calacademy.org/scientists/botany-collections (accessed on 30 August 2022)	CAS	19
21	Australia	Royal Botanic Gardens Victoria	https://www.rbg.vic.gov.au/science/herbarium/accessing-the-collection/ (accessed on 30 August 2022)	MEL	13
22	China	Lushan Herbarium, Jiangxi Province, Chinese Academy of Sciences	http://lbg.lsbg.cn:88/ (accessed on 30 August 2022)	LBG	8
23	China	Herbarium, College of Life Sciences, Sichuan University	/	SZ	6
24	Norway	Bergen Botanical Garden	https://www.uib.no/en/naturalhistory/148995/herbarium-bg (accessed on 30 August 2022)	BG	5
25	China	Herbarium, Yunnan University	/	YUKU	4
26	China	Herbarium of Northwest Institute of Plateau Biology, Chinese Academy of Sciences	/	HNWP	2
27	China	Herbarium of the Department of Biology, Fudan University	/	FUS	2
28	China	Fujian Normal University	/	FNU	2
29	China	Herbarium, Department of Biology, Peking University	/	PEY	1
30	China	Herbal Medicine Specimen Museum, School of Pharmacy, Peking University	/	PEM	1
31	China	Herbarium of Guangxi Institute of Botany, Chinese Academy of Sciences	/	IBK	1

Note: Some herbariums did not have official websites, and the related specimen records from these herbariums were accessed through GBIF (https://www.gbif.org/species/search, accessed on 30 August 2022) and iDigBio (https://www.idigbio.org/, accessed on 30 August 2022).

**Table 2:** The literature sources of collected specimen records.

No.	Literature
1	Plantae Chinenses Forrestianae: Catalogue of all the plants collected by George Forrest during his fourth exploration of Yunnan and eastern Tibet in the years 1917–1919.
2	Plantae Chinenses Forrestianae: Numerical catalogue of all the plants collected by George Forrest during his first exploration of Yunnan and eastern Tibet in the years 1904, 1905, 1906 (Nos. 1–1120).
3	Plantae Chinenses Forrestianae: Numerical catalogue of all the plants collected by George Forrest during his first exploration of Yunnan and eastern Tibet in the years 1904, 1905, 1906 (Nos. 1121–2757).
4	Plantae Chinenses Forrestianae: Numerical catalogue of all the plants collected by George Forrest during his first exploration of Yunnan and eastern Tibet in the years 1904, 1905, 1906 (Nos. 2758–4481).
5	Plantae Chinenses Forrestianae: Numerical catalogue of all the plants collected by George Forrest during his first exploration of Yunnan and eastern Tibet in the years 1904, 1905, 1906 (Nos. 4482–5099).
6	Plantae Chinenses Forrestianae: Catalogue of all the plants collected by George Forrest during his fourth exploration of Yunnan and eastern Tibet in the years 1917–1919: 001–148.
7	Plantae Chinenses Forrestianae: Catalogue of all the plants collected by George Forrest during his fourth exploration of Yunnan and eastern Tibet in the years 1917–1919: 147–406.
8	Plantae Chinenses Forrestianae: Catalogue of the plants (excluding *Rhododendron*) collected by George Forrest during his fifth exploration of Yunnan and eastern Tibet in the years 1921–1922.
9	Plantae Chinenses Forrestianae: Catalogue of the species arranged in alphabetical order.
10	Plantae Chinenses Forrestianae: Catalogue of the species arranged in natural orders.
11	Plantae Chinenses Forrestianae: Catalogue of the species arranged in natural orders (continued).

**Table 3:** Top 10 families with the most collected specimens.

No.	Family	Number of Specimens	Percentage
1	Ericaceae	7426	19.29
2	Rosaceae	2672	6.94
3	Asteraceae	2294	5.96
4	Primulaceae	2031	5.28
5	Orchidaceae	1640	4.26
6	Fabaceae	1288	3.35
7	Ranunculaceae	1200	3.12
8	Lamiaceae	799	2.08
9	Gentianaceae	682	1.77
10	Papaveraceae	595	1.55

**Table 4:** Top 10 families with the most collected species.

No.	Family	Number of Species	Percentage
1	Asteraceae	478	8.81
2	Rosaceae	335	6.17
3	Ericaceae	323	5.95
4	Fabaceae	230	4.24
5	Orchidaceae	229	4.22
6	Lamiaceae	205	3.78
7	Primulaceae	178	3.28
8	Ranunculaceae	173	3.19
9	Gentianaceae	116	2.14
10	Orobanchaceae	108	1.99

**Table 5:** Top 10 genera with the most collected specimens.

No.	Genus	Number of Specimens	Percentage
1	*Rhododendron*	425	17.10
2	*Primula*	419	3.83
3	*Cotoneaster*	373	1.09
4	*Gentiana*	363	1.08
5	*Saxifraga*	346	0.96
6	*Euonymus*	345	0.93
7	*Meconopsis*	339	0.89
8	*Pedicularis*	338	0.89
9	*Sorbus*	425	0.87
10	*Rubus*	419	0.87

**Table 6:** Top 10 genera with the most collected species.

No.	Genus	Number of Specimens	Percentage
1	*Rhododendron*	242	4.46
2	*Primula*	103	1.90
3	*Pedicularis*	85	1.57
4	*Gentiana*	70	1.29
5	*Saussurea*	67	1.23
6	*Saxifraga*	63	1.16
7	*Rubus*	51	0.94
8	*Cotoneaster*	51	0.94
9	*Corydalis*	41	0.76
10	*Ligularia*	39	0.72

**Table 7:** Top 10 species with the most collected specimens.

No.	Species	Number of Specimens	Percentage
1	*Rhododendron rupicola*	204	3.76
2	*Rhododendron beesianum*	145	2.67
3	*Rhododendron fulvum*	135	2.49
4	*Rhododendron primuliflorum*	128	2.36
5	*Rhododendron roxieanum*	121	2.23
6	*Rhododendron anthosphaerum*	119	2.19
7	*Rhododendron wardii*	113	2.08
8	*Rhododendron haematodes* subsp. *chaetomallum*	99	1.82
9	*Rhododendron adenogynum*	98	1.81
10	*Rhododendron racemosum*	97	1.79

**Table 8:** Species composition of collected specimens of *Rhododendron*.

Number of Specimens	Number of Species	Percentage
≥200	1	0.42
100–199	6	2.47
50–99	30	12.40
1–49	205	84.71
Total	242	100.00

**Table 9:** Species composition of collected specimens of *Primula*.

Number of Specimens	Number of Species	Percentage
≥40	4	3.88
30–39	9	8.74
20–29	12	11.65
10–19	27	26.21
1–9	51	49.52
Total	103	100.00

**Table 10:** Species composition of collected specimens of *Gentiana*.

Number of Specimens	Number of Species	Percentage
≥20	2	2.85
10–19	9	12.86
1–9	59	84.29
Total	70	100.00

**Table 11:** Species composition of collected specimens of *Cotoneaster*.

Number of Specimens	Number of Species	Percentage
≥20	8	17.78
10–19	6	13.33
1–9	31	68.89
Total	45	100.00

**Table 12:** Proportion of collected species during the seven expeditions.

Time	Number of Species	Percentage
First	1622	29.89%
Second	700	12.90%
Third	1991	36.69%
Fourth	1959	36.10%
Fifth	1127	20.77%
Sixth	918	16.19%
Seventh	399	7.35%
Others (Missing/Incorrect date)	3993	73.59%
Total	5426	100.00%

## Data Availability

Data will be made available on request.

## Supplementary Materials

The following supporting information can be downloaded at https://www.mdpi.com/article/10.3390/plants13101367/s1; Supplementary File S1: The list of species named after Forrest.
