# Supplementary material for: What Was George Forrest’s Plant Collection Journey like in China?
Source: Plants (Basel). 2024 May 15;13(10):1367. doi: 10.3390/plants13101367 (PMC11125649; doi:10.3390/plants13101367)
Supplement: Supplementary file 1 [file plants-13-01367-s001.zip › plants-3005786-supplementary.pdf]

plants

MDPI

The list of species name after Forrest

No.	Species Name	Publish Year
1	*Pleurogyne forrestii*	1907
2	*Meconopsis forrestii*	1907
3	*Primula forrestii*	1908
4	*Iris forrestii*	1910
5	*Aconitum forrestii*	1910
6	*Pedicularis forrestiana*	1911
7	*Codonopsis forrestii*	1912
8	*Caryopteris forrestii*	1912
9	*Chrysosplenium forrestii*	1912
10	*Cremanthodium forrestii*	1912
11	*Linnaea forrestii*	1912
12	*Abelia forrestii*	1912
13	*Buddleja forrestii*	1912
14	*Dumasia forrestii*	1912
15	*Acer forrestii*	1912
16	*Ajuga forrestii*	1912
17	*Scutellaria forrestii*	1912
18	*Galium forrestii*	1912
19	*Leptodermis forrestii*	1912
20	*Lespedeza forrestii*	1912
21	*Strobilanthes forrestii*	1912
22	*Rheum forrestii*	1912
23	*Hemerocallis forrestii*	1912
24	*Jurinea forrestii*	1912
25	*Sedum forrestii*	1912
26	*Habenaria forrestii*	1912
27	*Arenaria forresti*	1912
28	*Delphinium forrestii*	1912
29	*Allium forrestii*	1912
30	*Veronica forrestii*	1912
31	*Phlomis forrestii*	1912
32	*Hedychium forrestii*	1912
33	*Pleione forrestii*	1912
34	*Saxifraga forrestii*	1912
35	*Liparis forrestii*	1913
36	*Periploca forrestii*	1913
37	*Carex forrestii*	1913
38	*Cynanchum forrestii*	1913
39	*Pyrola forrestiana*	1913
40	*Braya forrestii*	1913
41	*Astragalus forrestii*	1913
42	*Hemipilia forrestii*	1913
43	*Parrya forrestii*	1914
44	*Phyllanthus forrestii*	1914
45	*Impatiens forrestii*	1915
46	*Camellia forrestii*	1916
47	*Dracocephalum forrestii*	1916

No.	Species Name	Publish Year
48	*Oreocharis forrestii*	1917
49	*Rhamnella forrestii*	1917
50	*Pentapanax forrestii*	1917
51	*Lysionotus forrestii*	1918
52	*Abies forrestii*	1919
53	*Artemisia forrestii*	1920
54	*Podocarpus forrestii*	1920
55	*Omphalogramma forrestii*	1920
56	*Pseudotsuga forrestii*	1920
57	*Petrocosmea forrestii*	1920
58	*Phoebe forrestii*	1921
59	*Garuga forrestii*	1921
60	*Staphylea forrestii*	1921
61	*Parasenecio forrestii*	1922
62	*Tsuga forrestii*	1923
63	*Viola forrestiana*	1923
64	*Leontopodium forrestianum*	1925
65	*Festuca forrestii*	1927
66	*Androsace forrestiana*	1927
67	*Chelonopsis forrestii*	1927
68	*Agapetes forrestii*	1927
69	*Microula forrestii*	1928
70	*Gentiana forrestii*	1928
71	*Serratula forrestii*	1928
72	*Manglietia forrestii*	1928
73	*Isodon forrestii*	1929
74	*Abies georgei* var. *smithii*	1929
75	*Acronema forrestii*	1930
76	*Notopterygium forrestii*	1930
77	*Jurinea georgei*	1933
78	*Saxifraga georgei*	1933
79	*Abies georgei*	1933
80	*Ilex forrestii*	1933
81	*Ilex georgei*	1933
82	*Rubus forrestianus*	1933
83	*Vernonia forrestii*	1933
84	*Heracleum forrestii*	1933
85	*Rhodoleia forrestii*	1933
86	*Meconopsis georgei*	1934
87	*Oreocharis georgei*	1934
88	*Aconitum georgei*	1934
89	*Inula forrestii*	1934
90	*Chirita forrestii*	1934
91	*Diospyros forrestii*	1934
92	*Saussurea georgei*	1934
93	*Incarvillea forrestii*	1935
94	*Gentiana georgei*	1936
95	*Syzygium forrestii*	1938
96	*Ranunculus felixii* var. *forrestii*	1939
97	*Begonia forrestii*	1939

No.	Species Name	Publish Year
98	*Berberis forrestii*	1941
99	*Abutilon forrestii*	1955
100	*Taraxacum forrestii*	1961
101	*Gueldenstaedtia forrestii*	1962
102	*Swertia forrestii*	1965
103	*Rhodiola forrestii*	1965
104	*Hypericum forrestii*	1970
105	*Koenigia forrestii*	1973
106	*Bulbophyllum forrestii*	1973
107	*Actinodaphne forrestii*	1974
108	*Roscoea forrestii*	1982
109	*Daiswa forrestii*	1983
110	*Maianthemum forrestii*	1986
111	*Utricularia forrestii*	1986
112	*Aristolochia forrestiana*	1989
113	*Cypripedium forrestii*	1992
114	*Allantodia forrestii*	1994
115	*Tylophora forrestii*	1995
